# Lifetime Observation of Cognition and Physiological Parameters in Male Mice

**DOI:** 10.3389/fnbeh.2021.709775

**Published:** 2021-09-01

**Authors:** Pia Kahnau, Anja Guenther, Marcus Nicolaas Boon, Janine Denise Terzenbach, Eric Hanitzsch, Lars Lewejohann, Vera Brust

**Affiliations:** ^1^Laboratory Animal Science, German Centre for the Protection of Laboratory Animals (Bf3R), German Federal Institute for Risk Assessment (BfR), Berlin, Germany; ^2^Department of Animal Behaviour, Bielefeld University, Bielefeld, Germany; ^3^Department for Electrical Engineering and Computer Science, Modeling of Cognitive Processes, Technische Universität Berlin, Berlin, Germany; ^4^Exzellenzcluster Science of Intelligence, Technische Universität Berlin, Berlin, Germany; ^5^Behavioral Phenotyping Unit, University of Osnabrück, Osnabrück, Germany; ^6^Animal Behavior and Laboratory Animal Science, Institute of Animal Welfare, Freie Universität Berlin, Berlin, Germany

**Keywords:** laboratory mice, cognition, IntelliCage, lifetime observation, resting metabolic rate

## Abstract

Laboratory mice are predominantly used for one experiment only, i.e., new mice are ordered or bred for every new experiment. Moreover, most experiments use relatively young mice in the range of late adolescence to early adulthood. As a consequence, little is known about the day-to-day life of adult and aged laboratory mice. Here we present a long-term data set with three consecutive phases conducted with the same male mice over their lifetime in order to shed light on possible long-term effects of repeated cognitive stimulation. One third of the animals was trained by a variety of learning tasks conducted up to an age of 606 days. The mice were housed in four cages with 12 animals per cage; only four mice per cage had to repeatedly solve cognitive tasks for getting access to water using the IntelliCage system. In addition, these learner mice were tested in standard cognitive tests outside their home-cage. The other eight mice served as two control groups living in the same environment but without having to solve tasks for getting access to water. One control group was additionally placed on the test set-ups without having to learn the tasks. Next to the cognitive tasks, we took physiological measures (body mass, resting metabolic rate) and tested for dominance behavior, and attractivity in a female choice experiment. Overall, the mice were under surveillance until they died a natural death, providing a unique data set over the course of virtually their entire lives. Our data showed treatment differences during the first phase of our lifetime data set. Young learner mice showed a higher activity, less growth and resting metabolic rate, and were less attractive for female mice. These effects, however, were not preserved over the long-term. We also did not find differences in dominance or effects on longevity. However, we generated a unique and valuable set of long-term behavioral and physiological data from a single group of male mice and note that our long-term data contribute to a better understanding of the behavioral and physiological processes in male C57Bl/6J mice.

## Introduction

Cognition comprises information processing mechanisms which enable decision making, i.e., perception, memory, and learning (McEwen, 2007; Gruszka et al., 2010; Roelofs et al., 2016). Cognitive abilities thereby enable adaptation to the social and physical environment of an individual. Especially complex or constantly changing environments are better coped with and exploited more successfully by species and individuals with an increased cognitive performance (e.g., Sol and Lefebvre, 2000; Lee, 2003; Dunbar and Shultz, 2007; Boucherie et al., 2019). Accordingly, a higher cognitive ability holds the potential to favor reproductive success and survival (Papini, 2002; Dukas, 2004). However, cognition is also associated with costs as the processing of information requires nervous tissue, which is energetically expensive to develop and maintain (Laughlin et al., 1998; Niven and Laughlin, 2008; Hollis and Kawecki, 2014). Adding up on this constitutive investment are the induced costs of building and maintaining particular memories (Snell-Rood, 2013). The trade-off between costs and benefits of cognition can elegantly be demonstrated by the fact that cognitive abilities are usually not maxed out under natural selection. Indeed, evidence from many different species shows that cognitive abilities can be substantially improved by artificial selection (e.g., Tryon, 1940; Brandes, 1988; Mery and Kawecki, 2002). In most habitats, however, it is generally assumed that natural selection prevents a permanent improvement of cognitive abilities due to trade-offs with other fitness related traits (Buchanan et al., 2013). Thus, an evolved species has a cognitive range within which an individual must incur the corresponding physiological costs depending on the energetic expenditure of brain activity. Studies linking cognition directly to physiological, reproductive, or survival traits are limited (e.g., Cole et al., 2012; Huebner et al., 2018). Therefore, we still know little about how these trade-offs actually shape cognitive abilities or, on an individual level, affect how an individual uses its cognitive abilities. Here, we follow a cohort of male mice throughout life with the aim to study the effects of a cognitively demanding life on different aspects of physiology, reproduction, and survival.

By provisioning an environment which constantly held new cognitive challenges to some but not all of the mice, we wanted to test for the consequences of such different lifestyles. Based on the above mentioned trade-offs, cognitively stimulated mice might have a reduced or slower growth and in addition or alternatively a higher energy turn-over as compared to non-stimulated mice. The energy consumption of an individual can be determined by measuring its metabolic rate, i.e., oxygen uptake and carbon dioxide release, indicating how much energy is produced by aerobic respiration (Brown, 2004). While a direct influence of learning performance or use of cognitive abilities on metabolic rate to our knowledge has not yet been investigated, the metabolic rate in this study was measured four times throughout the mice’s life. We wanted to investigate a possible relation between different learning environments suggesting a higher metabolic rate in males which were cognitively stimulated repeatedly throughout their lives.

Besides these possible physiological contrasts between the differently stimulated mice, we assessed an aspect of reproductive success. Only male mice were included in the study and we tested them twice in a female choice task where potential mates were allowed to freely choose to spend time in close proximity with individuals of the different testing groups. Some studies in insects and vertebrates have shown that females choose mates with better cognitive skills reflected in males’ courtship behavior, performance in foraging or in diet-dependent morphological traits [reviewed in Boogert et al. (2011)]. Additionally, males of species with a complex and competitive sexual environment may have to process complex sensory information and display learned abilities in courting females (Byrne and Rice, 2006; Dukas, 2006; Griffith and Ejima, 2009). In an experimental evolution study in fruit flies cognitive performance of males declined under the absence of sexual selection (Hollis and Kawecki, 2014). Consequently, we might suggest an impact of the different learning environments applied to our tested mice in relation to their attractiveness toward females. Still, as their potential differences in cognitive abilities are not directly on display in our testing context, it is difficult to predict whether the females are able to include these traits in their decisions. In addition to female choice, male-male competition is a core principle of sexual selection. In order to measure whether or not the cognitive stimulation affected intrasexual competition, we performed direct observations of aggressive behavior within the social groups of male mice.

Measuring longevity is a straightforward way to test if the learning environment has fitness consequences. Under natural conditions, longevity may be directly influenced by differences in foraging success or predator avoidance (e.g., Madden et al., 2018). Accordingly, higher cognitive abilities have repeatedly been found to be positively linked to survival in the wild (see Morand-Ferron, 2017 for an overview). However, under captive conditions, differences in predator avoidance or foraging success are unlikely to occur because animals are usually protected from predation and provided with food *ad libitum*. Nevertheless, the link between physiological condition and cognitive function could affect longevity even under captive conditions, especially if differences in early environments influence physiological and cognitive development through phenotypic plasticity (Pravosudov et al., 2005; Loi et al., 2017). Accordingly, replicate populations of fruit flies selected for an improved learning ability have shown a pronounced reduction in longevity and conversely, lines selected for extended longevity showed a reduction in learning ability (Burger et al., 2008). Our tested mice were kept over their whole lifespan enabling us to directly test for an effect of the applied learning treatment on longevity. In accordance with former laboratory studies, we expect a reduced lifespan in mice kept under cognitively stimulating conditions as compared to the non-stimulated mice. Besides this direct measurement of longevity, we investigated the telomere length of the mice at a later stage in life. Telomeres comprise of repeated and non-coding DNA strands forming the ends of each chromosome and have been linked to rates of aging and age-related diseases in aging human and non-human individuals (Von Zglinicki, 2002; Brouilette et al., 2003; Benetos et al., 2004; Martin-Ruiz et al., 2006). In addition, several studies such as for example by Yaffe et al. (2011) showed that telomere length can also serve as a marker for cognitive aging in humans with shorter telomeres going along with reduced cognitive abilities. While the effects of stressful environments on telomere length and cognitive decline are well described, it is not yet known if an environment that imposes elevated cognitive processes like applied in our study can affect telomere lengths.

Considering all the above, we hypothesize that cognition in male mice throughout life affects physiology (body mass development, metabolic rate), sexually selected traits (competitive ability, male attractiveness), and longevity (measured by actual survival and telomere length) across life. A unique feature of our study is that mice were kept in groups in a home-cage based test apparatus, the IntelliCage (IC, New Behavior) system. This way, individual testing paradigms could be applied to each animal enabling us to form social groups that contained both, learner and non-learner mice. Previous studies show that mice are able to solve learning tasks within the IC and also other parameters such as activity patterns can be measured within the system (Galsworthy et al., 2005; Mechan et al., 2009; Krackow et al., 2010; Endo et al., 2011; Voikar et al., 2018). Home-cage based test systems offer the advantage of testing animals without daily interference of researchers and in accordance with the animals natural activity phases over a long period of time [reviewed in Voikar and Gaburro (2020)].

Before going into further detail, we wish to emphasize that this study comprises data from three initially independent Master theses which all focus on the costs of cognition. We reverted to the same group of mice in all theses which allowed us to compile a unique lifetime observation data set presented in the current study. In this set-up, we consequently had to face slight variations in experimental procedures due to the changing experimenters whereas the same overarching research question allows the data to be presented as a single long-term study with three consecutive phases. In summary, we present a unique and comprehensive lifetime observation of 48 male mice, which to our knowledge has no comparison in the present literature record.

## Materials and Methods

### Animals and Housing Conditions

48 male C57BL/6J mice (Charles River, Sulzfeld, Germany) arrived at Osnabrück University at the age of 21 days. The mice were randomly separated in four housing groups, 12 mice per cage, which were kept stable over the lifetime of the mice. One day after arrival, the mice received an RFID transponder (radio frequency identification ISO FDX-B 2.12 × 12 mm, Planet ID GmbH, Essen, Germany), which was implanted subcutaneously into the neck area under isoflurane anesthesia. Each social group of 12 mice was further randomly assigned to one of three treatment groups: The learner mice (L), which had to solve various cognitive tasks, the non-learner (NL), which had no tasks to solve, and the equipment control group mice (EC), which also had no tasks to solve, but were exposed to handling similar to that of the L mice during testing. For visual identification, each mouse was assigned a unique two-color code which was applied to the tail skin with lacquer painting pens (edding 750).

During their growth phase up to an age of 73 days, body mass was measured every 3 days. Adult mice were weighted weekly during the process of cage cleaning up to an age of 757 days, except for a non-experimental phase between 192 and 302 days of age. During handling (tail-handling), the tail color-codes were additionally renewed if necessary. The mice were kept at 22 ± 2°C at 56 ± 15% humidity. The dark/light cycle was 12 h each, with light hours between 8:00 am and 8:00 pm. Pellet food (Altromin International, 1314) was available *ad libitum* at all times. At the age of 325 days, the mice moved to another animal facility within the university. The housing conditions were similar but with an additional half hour of sunrise simulation and the last half hour of the light cycle simulating sunset. Within these facilities mice were kept in different cage types throughout their life as described in detail below. Mice were kept in standard home-cages after the last experiment until they died a natural death. However, to keep animal welfare standards mice were euthanized as soon as signs of pain or suffering were detected.

### IntelliCage

**FIGURE 1:**
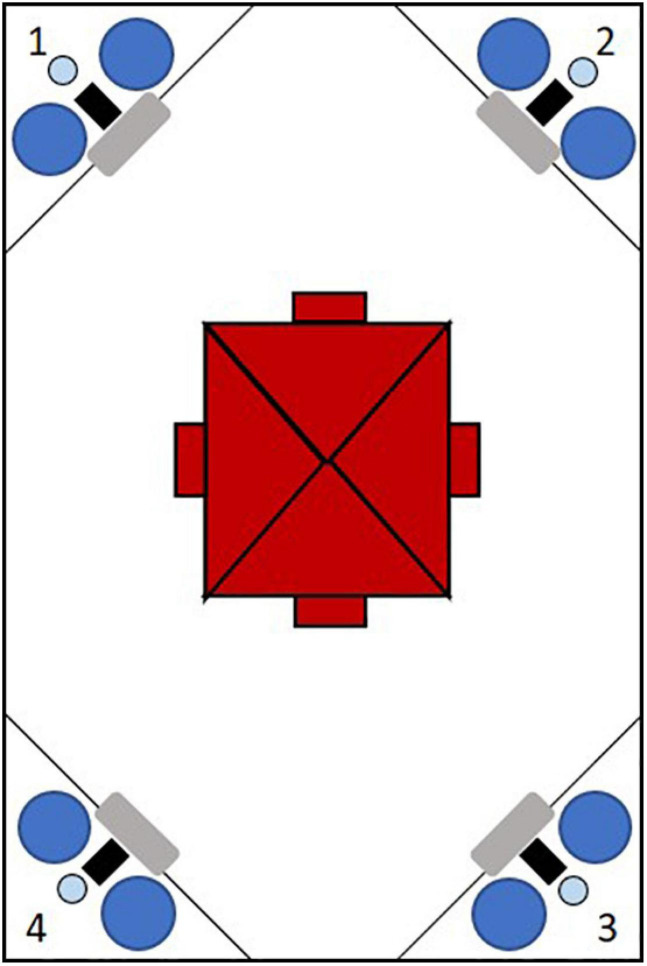
Schematic figure of an IntelliCage from the top with four conditional corners marked by numbers. Each corner contains a radio frequency identification antenna (gray), a presence-sensor (black), an airpuff valve (light blue) and two water dispensers (dark blue). In the cage center four shelters (red) are arranged under the food rack.

The IntelliCage (IC, NewBehavior) is a home-cage in which various cognition tasks can be applied to individual animals with minimal invasion by experimenters. Each IC contained bedding (Allspan, Olympia, 2 cm high), paper as nesting material and four red mouse houses (“TheMouseHouse;” Tecniplast) for shelter. The houses were placed directly under a central feeding rack. Water was available in eight dispensers arranged in the four cage corners (Figure 1). Each corner is only accessible to one mouse at a time. Each corner contains a presence sensor, one RFID antenna and an airpuff valve for mild punishment (0.5 bar). Access or denial to water can be granted to individual animals identified via an implemented RFID sensor, enabling individual operant conditioning in group housed animals. Each corner contains a nosepoke-sensor and a door per water dispenser. The doors can be opened by a nosepoke detected by the nosepoke-sensor. By assigning mice access to individual corners or water dispensers at certain times or in certain orders, tasks of different levels of difficulty could be applied. Nosepokes at non-rewarded dispensers were punished with a 1 s and 0.5 bar airpuff in certain conditions.

During their stays in the IC, different programs of various difficulty were applied to the L mice allowing and denying access to different corners or water dispensers (Table 1). As water was only offered in the cage corners, we assume that the mice were primarily entering the corners for fluid intake. Accordingly, attempts to visit corners and drink at certain water dispensers reflects the animals’ foraging effort. Therefore, we use the term “foraging behavior” to describe the behavior of corner visiting for getting access to water. The IC tasks got more and more difficult over time including patterns in which the rewarded corner or side within a corner changed after each successful drinking event, like for example to the opposite corner or in a clock- or anticlockwise manner. The difficulty of the IC tasks was increased by allowing the L mice access to water in all four corners, but the L mice received an airpuff in addition to water in three (incorrect) corners. Only in one (correct) corner, drinking was possible without receiving an airpuff. But drinking within the correct corner on the incorrect side leads to a corner change (for example clock- or anticlockwise). If the L mice drank on the correct side within the correct corner, the correct corner did not change (see Supplementary Material for full details on all IC cognition tasks). Some of the IC tasks were repeated, adding up to 51 learning tasks applied to the L mice in the IC. Both the NL and EC mice had access to water at all times but were punished by an airpuff if they stayed for longer than 15 s in a single corner to avoid a mouse occupying a corner and blocking the access for other individuals for too long. In addition, while the NL and EC mice had 15 s to drink, the L mice in the different IC tasks had 8–10 s to drink water within the correct corner or on the correct side. In order to drink again, all mice had first to leave the corner and re-enter it or visit another corner. In total, the mice were kept in the ICs in three phases of different lengths (Table 1, IC phase 1 = 32 tasks, IC phase 2 = 16 tasks, and IC phase 3 = 3 tasks). Even though the L mice had less time within the correct corner to drink and were not able to drink in all corners without punishment relative to the NL and EC mice, we compared the foraging behavior of the three treatment groups with each other. This was carried out by analyzing the number of corner visits that were performed (per week) by the mice. A visit was evaluated as soon as a mouse entered a corner, the RFID antenna registered the RFID transponder of the mouse and at the same time the presence sensor registered the presence of the mouse.

**TABLE 1:** Learning IntelliCage tasks assigned to learner mice within the IntelliCage.

IC Program	test duration (days)*	IC phase
Cornerlearning 1	14	1
Shuttling 1	12	1
Clockwise 1	11	1
Anticlockwise 1	14	1
Clockwise 2	9	1
Sidelearning	14	1
Cornerlearning 2	13	1
Shuttling 2	7	1
Clockwise 3	9	1
Anticlockwise 2	19	1
Clockwise 4	6	1
Anticlockwise 3	13	1
Clockwise 5	10	1
Complexclockwise 1	10	2
Complexshuttling	14	2
Complexanticlockwise 1	12	2
Clockwise 6	3	3
Complexclockwise 2	2	3
Complexanticlockwise 2	8	3

*See Supplementary Material for exact times in hh:mm:ss.

### Standard Home-Cage

**FIGURE 2:**
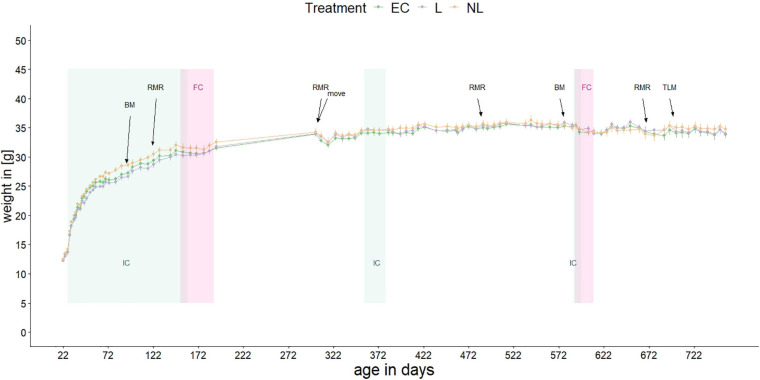
Body mass of mice over their lifetime. Shown are the mean body masses of the three treatment groups. At 305 days of age, the mice moved to another animal facility. Repeated experiments were conducted: IC, IntelliCage; FC, female choice test; BM, Barnes maze test; RMR, resting metabolic rate; TLM, telomere length measurement. When not in the ICs, the mice were kept in standard home-cages within their social groups.

Mice were kept in type IV Macrolon cages (59 cm × 59 cm × 20 cm) per social group. Food and water were available *ad libitum*. Each home-cage contained two red houses, a plastic tube, and paper for nesting and bedding. No tests were applied directly in the home-cage, but mice were transferred to different tests over short time periods during their stays in the home-cage (for an overview of the timeline see Figure 2 and Supplementary Table 1).

### Barnes Maze Test

The Barnes maze test (BM) was carried out to test for spatial learning performance of L mice outside the IC. It uses the natural aversion of mice to open and exposed areas (Crawley, 1985) and measures how many errors they make to escape from such an area over the course of multiple trials. We used a round gray plastic platform of 100 cm diameter mounted about 120 cm above the ground as the exposed area. The platform contained 12 holes of 4 cm diameter evenly distributed along its edges, which could be closed via opaque black plexiglass lids. One hole could be connected via a 4 cm diameter PVC tube to a cage (Makrolon type III, filled with bedding transferred from the cage the mouse was housed in) placed under the platform. Visual cues (a bottle, a cloth, a metal stand, and a box) arranged around the platform served as spatial orientation cues. The test was performed during the light phase at 22 ± 1°C and with 54 ± 4% humidity in the experimental rooms. The illumination on the platform was 10–20 lux. Prior to each trial, the platform was cleaned with ethanol (70%).

For each trial, a single L mouse was placed in a start-cylinder (diameter: 10.5 cm, high: 21 cm) in the middle of the platform for 1 min. After this acclimatization phase, the start-cylinder was removed and the trial started. The number of attempts to enter covered holes to escape from the platform were evaluated from video recordings (Logitech HD webcam, Software: VirtualDub Version 1.9.11) to investigate learning performance. The BM test was performed twice at the age of 92 and 573 days. In the first run each trial lasted 5 min. Each mouse performed ten trials, two trials per day with an inter-trial interval of 30 min on five consecutive days. In the second run the test was performed in a different room and with different positions of the escape hole and the spatial cues surrounding the platform, but the same apparatus with the same illumination conditions was used. For the second run, each mouse performed six 3-min trials, two trials per day with an inter-trial interval of 30 min over three consecutive days. In both runs, if a mouse did not find the open hole within the given time, it was gently guided to the escape hole by the researcher’s hand. To control for a possible handling-effect, each L mouse was assigned to an EC mouse. After the L mouse finished the BM test, the assigned EC mouse was placed on the platform of the maze for exactly the same time it took the L mouse to find the open hole. Contrary to the L mouse, the matched EC mouse could not escape from the platform, as all 12 holes were closed with the black opaque plexiglass.

For further cognitive stimulation outside the IC, a T-maze test, a tone conditioning test (carried out during the first IC phase) and a labyrinth experiment (after the third IC phase) were carried out. However, the results of these tests were not sufficiently conclusive to be included in this work due to methodological problems (e.g., we only know now why the T-Maze did not work: Habedank et al., 2021). Nevertheless, it is important to mention that the control group had other experiences outside the home-cage besides the BM test (for more information about the test procedure see the supplements).

### Female Choice Tests

To test for possible differences of attractiveness to females between the three treatment groups of mice, the males were subjected to two female choice tests (FC), one at an age of 152 days (shortly after IC phase 1) and a second one at an age of 590 days (several months after IC phase 3). Females used in the first test were C57BL/6J mice naive to cognitive experiments (*N* = 16) and 152 days old. Females in the second test were the first generation offspring of C57BL/6J X BALB/C and subjected to cognitive tests including an IC phase themselves prior to the choice tests (*N* = 12, each female conducted a maximum of two choice tests). At the time of testing the females were 304 days old. The estrus status was not determined. Nevertheless, we must note that the estrus status may affect the female’s behavior. In each test, one female was introduced into a type III Makrolon cage filled with fresh bedding for a 1 h habituation time. Accordingly, three males, one of each treatment group were randomly placed in three similar cages divided in half by a perforated clear plexiglass wall. The female’s cage was connected to the three empty half compartments of the males’ cages via PVC tubes. The location of the female and the time spent with each male over the course of 24 h was automatically recorded using light barriers installed in each tube.

### Observations of Agonistic Behavior

To test whether the outcome of agonistic encounters between mice within each social group was influenced by the treatments, live observations on fighting behavior were conducted. Mice within the social groups in general lived rather peacefully together throughout their whole lives. At no time any mouse had to be removed from a group due to social incompatibility. The only times when agonistic behaviors occurred more frequently were during the weekly cage cleaning events, when mice were transferred to clean cages. Cage cleaning and accordingly live observations of agonistic behavior took place once a week between 08:30 am and 11:30 am. The mice of one social group were placed in random order into an observation cage containing only fresh bedding, food, and water. One minute after all mice were placed in this cage, they were observed by an experimenter for 30 min. Within this time, the mice usually calmed down and agonistic behavior almost ceased to occur. The experimenter noted down the IDs of mice involved in fights as well as who won and lost each fight. The behavior *fight* began as soon as two mice began to circle one another with body contact. During a *fight*, the mice pushed one another with their paws and bodies or bit, sometimes even pushing the opponent with its back to the ground. *Fights* could be accompanied by vocalizations, too. A *fight* ended when one of the two opponents turned its head away from the other. The loser mouse was the one which turned away first. Fights between three or more mice were not included in the data set, as identification of individuals as well as the determination of winners and losers were less straightforward. Observations of agonistic behaviors were done when the mice were between 373 and 759 days old.

### Resting Metabolic Rate

To test whether the different treatments of mice led to differences in their metabolism, respirometry measurements were repeatedly taken throughout their life. Respirometry is a method for determining the total energy turnover of an organism. The release of carbon dioxide is determined in relation to oxygen absorption. This allows determining the metabolic rate of an organism. The resting metabolic rate (RMR) measurement was carried out at the Department of Animal Behavior at Bielefeld University. The measurement was performed at 20 ± 1°C under low light conditions in an open flow system by measuring the oxygen consumption and carbon dioxide production with a continuous inflow (45 l/h, Mass Flow Meter FM-360, Tylan Corp., Torrance, CA, United States) of external fresh air. External air was transferred under ambient pressure to two transparent Plexiglas measuring chambers (14 cm × 20.5 cm × 14 cm), which were placed in a climate chamber (Rubarth Appaerate, Laatzen, Germany) and contained paper (for excretion absorption) but no water or food. Both chambers were located in a way that animals could not see, hear, or smell each other. For drying, the air was first pumped to two cooling devices (M&C Cooler, Ratingen, Germany) and then transferred to a molecular sieve. The oxygen consumption and carbon dioxide production were analyzed by an O_2_ analyzer (Oxzillar FC, Sable Systems, Henderson, NV, United States) and CO_2_ analyzer (Maihak AG, Hamburg, Germany). As a control, we compared dried outside air against the carbon dioxide and oxygen concentrations measured in the outflow of the metabolic chambers, in which the test animals rested. An initial control period of 10 min was used to assure the stability of the system. Over a period of 2.5 h, each animal’s oxygen consumption and carbon dioxide production were measured across six periods of 10 min each. Between measurements, 1-min control intervals were interspersed to allow correction for system drifting if necessary. The specific RMR (KJ/(d^∗^kg)^–1^) was calculated from the 3-min interval with the lowest, stable oxygen consumption throughout the measurement periods. Within the apparatus, two animals were measured simultaneously between 08:30 am and 05:00 pm. The evening before the measurement the mice were separated in type III Macrolon cages (with nesting and bedding, water and food from the home-cage) to habituate them to being separated during measurement. Directly before and after the measurement, body mass was measured. After RMR measurement, the mice were placed back into their home-cages. The RMR was measured four times, at an age of 138, 308, 482, and 665 days.

### Telomere Lengths Measurement

To investigate whether repeated cognitive stimulation affects the length of telomeres, from all 44 mice still alive (15 L mice, 14 NL mice and 15 EC mice) at an age of 699 days, a blood sample was taken from the tail vein. Previous studies showed a link between telomere length and aging, age-related diseases or cognitive aging (Von Zglinicki, 2002; Brouilette et al., 2003; Benetos et al., 2004; Martin-Ruiz et al., 2006; Yaffe et al., 2011). By repeated cognitively stimulating the L mice, we assumed a difference in telomere length between the three treatments. Therefore, in our study telomere length should serve as a longevity marker (in addition to the classical survival analysis). DNA was extracted out of the leukocytes according to the instructions of the UltraClean^®^ Blood DNA Sample Kit (Non-Spin, MO BIO Laboratorie, Inc.) and used in a telomere real time quantitative polymerase chain reaction (RT qPCR) performed with a BioRad PCR system consisting of the BioRad CFX96^TM^ RealTime System as optical reaction module and BioRad C1000 Touch^TM^ Thermal Cycler following Cawthon et al. (2003) and Callicott and Womack (2006). From each sample DNA and from a control DNA (single copy gene 36B4), three replicates were prepared. The dilutions of the sample DNA were: 100, 20, 4, 0.8, and 0.16 ng/5 μl. The dilutions of the control DNA were: 20, 4, 0.8, 0.16, and 0.032 ng/5 μl. In addition, a zero-control (Null Template Control-NTC) was prepared with three replicates. 10 μl GoTag^®^qPCR Master Mix, 1.8 μl telg-Primer, 1.8 μl telc-Primer and 1.4 μl PCR-water were added to the DNA samples. 10 μl GoTag^®^qPCR Master Mix, 1.8 μl forward-Primer, 1.8 μl reverse-Primer and 1.4 μl PCR-water were added to the control samples. The relative telomere length was then determined from the C_T_ value of the sample DNA and the C_T_ value of the control DNA.

### Data Analysis

The following data analysis and visualization was performed with R (R Core Team, 2020; version 3.4.2) and Python (version 3.8), using the panda package (version 1.2.3).

#### Body Mass Measurement

For body mass analysis four linear mixed effects models (package nlme, Pinheiro et al., 2020) with weight (log transformed), housing condition (housed in the IC vs. housed in the home-cage) and age set as fixed effects were calculated for each of the three IC phases and in the home-cage. Animal ID served as a random effect. Model assumptions were checked visually by Q–Q plots and by plotting fitted versus residual values. To test for effects between treatment groups within housing conditions, pairwise *post hoc* comparisons were conducted (package emmeans, Lenth, 2020).

#### IntelliCage

For each task in the IC, the performance of the L mice was determined by calculating the ratio of successful trials over all trials. For experiments where the task depended on a corner condition (i.e., choosing the correct corner), visit events were used as a measure for a trial, whereas for experiments where the task depended on a side condition (i.e., choosing the correct side within a corner), nosepoke events were used as a measure for a trial. A criterion of cognition was met when the proportion of successful trials within a task for a mouse was higher than 1.25 times chance level (e.g., 31.25% correct for cornerlearning), demonstrating that there was an understanding of the task.

The foraging behavior (number of corner visits) in the IC was analyzed in a Poisson GLMM (package lmerTest, Kuznetsova et al., 2017) including treatment group and phase as fixed effects and animal ID as random effect. To test for effects between treatment groups and between phases, a pairwise *post hoc* analysis with Tukey adjustment was conducted (package emmeans). Due to technical issues of the first part of the first IC phase, the changes in task objective during “cornerlearning 1” did not consistently change at the specified timestamp. Instead, for some L mice the correct drinking corner changed at a different moment than for other mice. Therefore, we decided to ignore the first 30 min of each “cornerlearning 1” task.

#### Barnes Maze Test

The two runs of the BM were evaluated separately, as they differed in execution (first run: 10 trials of 5 min each, second run: 6 trials of 3 min each). The number of errors was analyzed in a Poisson GLMM (package lme4, Bates et al., 2015) including errors and trial as fixed effects and animal ID as random effect. To test for effects between trials, a pairwise *post hoc* analysis with Tukey adjustment was conducted (package emmeans). Residuals of the model were visually inspected for homogeneity of variances and normal distribution by using QQ plots.

#### Female Choice Tests

Including only females which choose one of the males (i.e., which spent less than 50% of time alone in the starting cage and/or had less than 5% difference in the amount of time spent with their first versus second choice male) in the analysis, it comprised of 14 (out of 16) choices in the first test and 12 (out of 16) choices in the second test, respectively. For these females, we calculated the percentages of time spent with each specific male from the whole time a female spent in cages with males. These percentages were then analyzed in a GLMM (package lme4) including male treatment group, cage orientation within the test set-up and male mass rank (1–3) within the trio of males as fixed effects and female identity as random effect. In addition, we ran a model in which male mass rank was replaced by mean-centered male body mass to test for the effect of male body mass within the given set of males for each test and, more generally, across all tested males. When the model indicated a significant main effect of male treatment or cage, we conducted a *post hoc* comparison on the fixed effect using the false-discovery rate to adjust *p*-values (package emmeans).

#### Observation of Agonistic Behavior

The number of fights each mouse was involved in during the whole time period of life observations was calculated along with the number of fights this individual won. For each of the two variables, we ran a GLM (package lmerTest) to test the effect of treatment, cage and mean weight over the observation period.

#### Resting Metabolic Rate

For RMR analysis a linear mixed model (package lme4) was carried out with RMR, treatment and RMR test run as fixed effects and an interaction of treatment and RMR test run. Animal ID served as a random effect. If the model indicated a significant effect of treatment or test run, we conducted a pairwise *post hoc* analysis with Tukey adjustment (package emmeans).

#### Telomere Length

By the time of blood collection for telomere length measurement (699 days of age), 44 out of 48 mice were still alive (15 L mice, 14 NL mice, 15 EC). A linear model was conducted to analyze a possible effect of treatment on telomere length. Treatment was set as fixed effects (continuous effects). To obtain normally distributed data, the relative telomere length was transformed by logarithms.

#### Survival Analysis

For survival analysis, a Cox proportional-hazards model (Coxph) was calculated (package survival; Therneau and Grambsch, 2000). Treatment and telomere length were included as fixed effects in this model.

## Results

### Description of Learning Outcomes

#### IntelliCage

**FIGURE 3:**
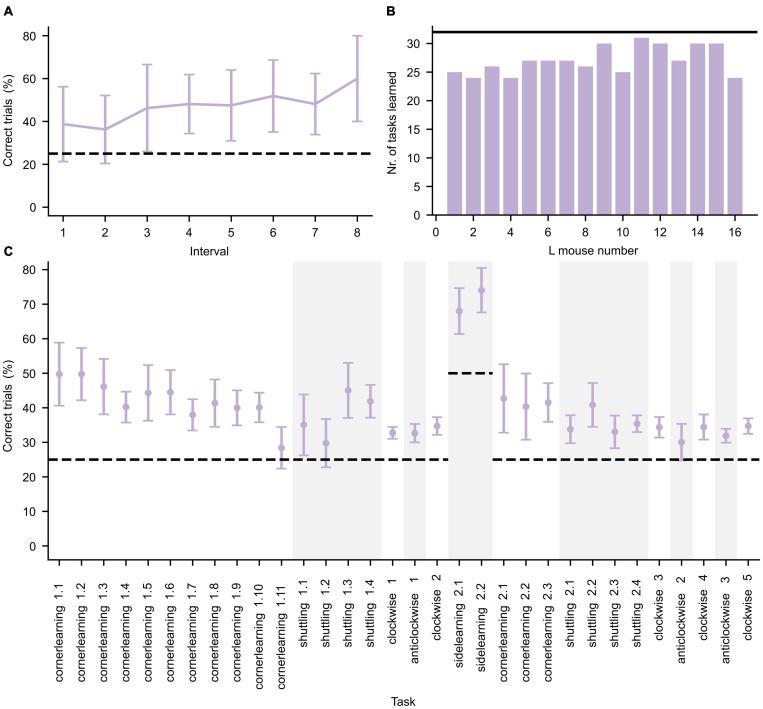
Performance during the first IntelliCage phase. **(A)** Average performance (±standard deviation, *n* = 16) of all L mice for the first day of the cornerlearning task, divided in intervals of 10 trials per interval. Since the least active mouse performed less than 90 trials, only the first eight intervals are shown. The increase of performance over time reflects learning in the mice. **(B)** Number of tasks for which the L mice successfully achieved the learning criterion. The black horizontal line represents the total number of tasks. **(C)** Average performance (±standard deviation, *n* = 16) per learning task. Changes in task type are separated by alternating white and gray background color. The learning tasks are represented in chronological order. The dashed lines in panels **(A,C)** represent the chance level of correct performance in each task.

The L mice were cognitively trained during three IC phases requiring them to perform various learning tasks to get access to water as reward (see Supplementary Information). To confirm that the mice were indeed cognitively stimulated by the tasks, their performance on each task was investigated. The mice performance was evaluated by calculating the percentage of successful trials over the entire task duration. To illustrate an example of the learning behavior of the group and the magnitude of inter-individual variability of the mice, the learning curve for the first learning task (cornerlearning 1) is shown in Figure 3A. A summary of task performance for the first IC phase per mouse is given in Figure 3B. The learning criterion was set to 1.25 × chance level. If a mouse did not reach this threshold, we assumed this mouse did not understand the underlying task’s structure. Each mouse was able to learn at least 24 out of the 32 tasks, demonstrating that the mice were overall cognitively stimulated. The average performance of the mice per task is summarized in Figure 3C for the first IC phase.

**FIGURE 4:**
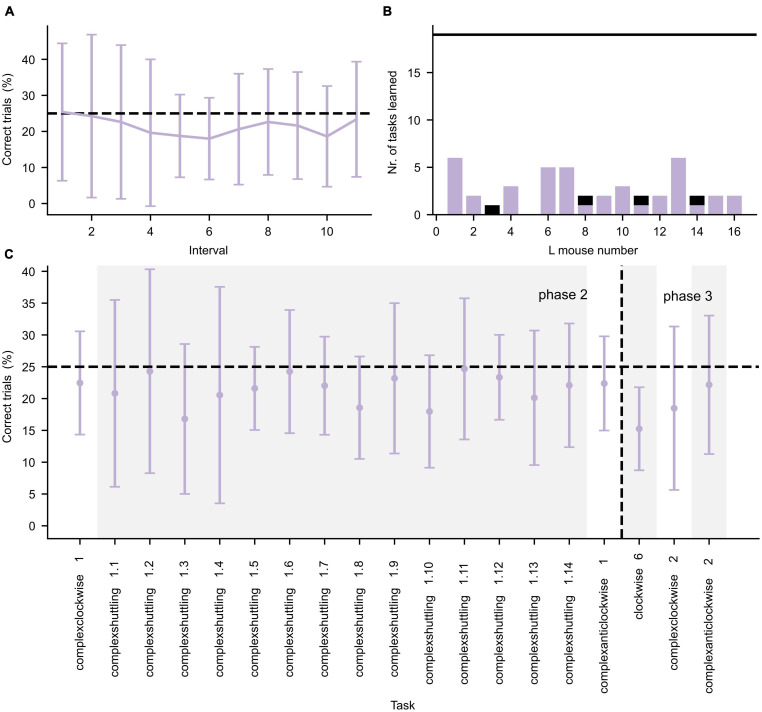
Performance during the second and third IntelliCage phase. **(A)** Average performance (±standard deviation, *n* = 16) of all L mice for the first 550 trials of the Complexclockwise 1 learning task, divided in intervals of 50 trials per interval. **(B)** Number of tasks for which the L mice successfully achieved the learning criterion. The black horizontal line represents the total number of tasks. The purple and black bars represent successful tasks during phase 2 and 3, respectively. **(C)** Average performance (±standard deviation, *n* = 16) is given by the error bar data points. Changes in task are separated by alternating white and gray background color. The learning tasks are represented in chronological order. The horizontal dashed lines in both panels **(A,C)** represent the chance level of correct performance in each task. The vertical dashed line in panel **(C)** represents the separation between IC phase 2 and 3.

During the second and third IC phase, more complex tasks were presented, which are shown in Figure 4. Again, the learning curve for the first learning task (complexclockwise 1) is shown in detail in Figure 4A. In contrast to the learning performances during the first IC phase, during the second and third IC phases the mice often did not achieve the learning criterion (Figure 4B). As illustrated in Figure 4C, the mice performed roughly at chance level for the tasks.

**FIGURE 5:**
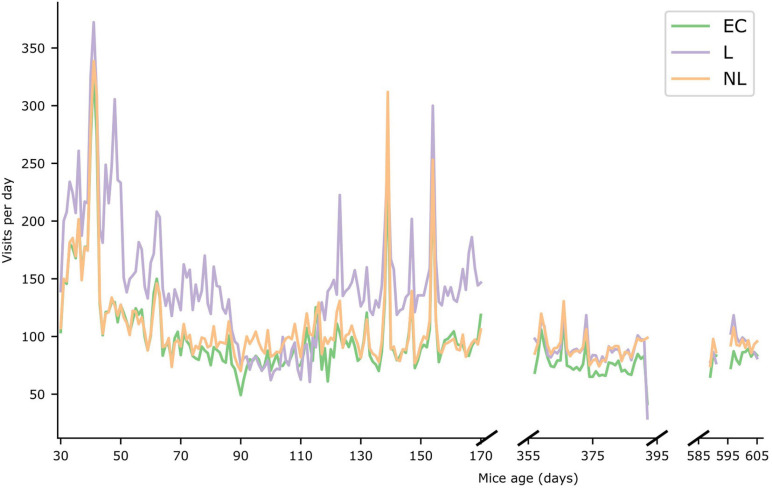
Foraging behavior. Number of visits per day during the three IntelliCage phases. The X-axis represents the age of the mice (in days). The average value of the three different treatment groups are shown for the three IntelliCage phases.

In Figure 5 the daily foraging behavior of the three treatment groups in the IC are shown (see Supplementary Figure 1 for the daily number of licks per treatment group). During the first phase, the foraging behavior of the L group was significantly higher (GLMM, *p* < 0.001) than the other groups. During the second and third phase the foraging behavior of the L and NL groups was almost identical (GLMM, *p* > 0.05), whereas the difference in foraging behavior between the EC and L group and EC and NL group were significantly different (GLMM, *p* = 0.0025 and *p* < 0.0014, respectively, for phase 2, *p* = 0.03 and *p* = 0.029, respectively, for phase 3). Furthermore, all treatment groups were significantly less active in subsequent phases (GLMM, *p* < 0.001) except for the NL treatment group phase 2 and 3 (*p* > 0.05).

#### Barnes Maze Test

**FIGURE 6:**
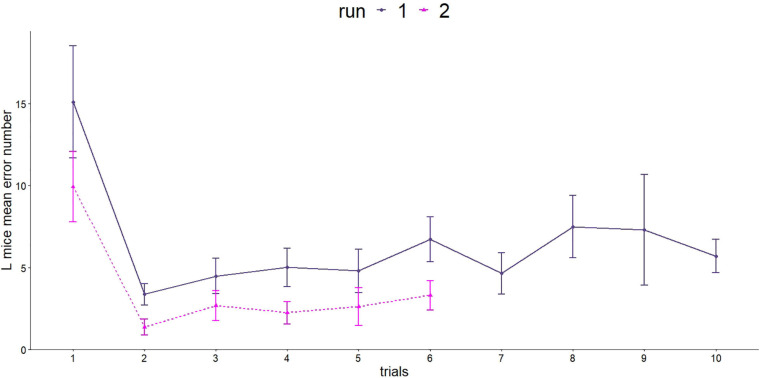
Number of errors made in the Barnes maze test. Since the two runs were carried out differently (number of trials and length of trials), a separate statistical evaluation was carried out. First run, dark purple line: *n* = 16; age = 92 days; second run, purple line: *n* = 16, age = 573 days.

Spatial orientation and memory were investigated using the Barnes maze twice. L mice were able to learn and improve their performance in both runs (Figure 6). In the first run, the mice made significantly more mistakes in the first trial compared with all following trials (GLMM, *p* < 0.001, *post hoc* comparison).

The second run showed a similar picture for the number of errors made. The steepest decrease was observed between trial 1 and 2 (GLMM, *p* < 0.001).

### Effects on Physiology/Body Maintenance

#### Body Mass

Body mass of the mice was measured between 21 and 757 days of age with a gap between day 192 and 302 (Figure 2). At the beginning of the study the mean body mass was 12.37 g, at the end of the measurement 34.12 g. When the mice were housed in the IC’s for the first time, the body mass differed between L and NL mice (GLMM, *post hoc* comparison, *t* = −2.71, *p* = 0.025) while there was no difference between L and EC mice (GLMM, *post hoc* comparison, *t* = 1.08, *p* = 0.531) or between NL and EC mice (GLMM, *post hoc* comparison, *t* = −1.63, *p* = 0.243). When the mice were housed in the IC for the second and third time as well as in their home-cages, no treatment effect on weight was detected (GLMM, *post hoc* comparison, IC phase 2: EC vs. L: *t* = −0.88, *p* = 0.659; EC vs. NL: *t* = −0.70, *p* = 0.764; L vs. NL: *t* = −0.17, *p* = 0.984; IC phase 3: EC vs. L: *t* = −0.27, *p* = 0.960; EC vs. NL: *t* = −0.24, *p* = 0.969; L vs. NL: *t* = −0.03, *p* = 1; home-cage: EC vs. L: *t* = −0.03, *p* = 1; EC vs. NL: *t* = −0.91, *p* = 0.640; L vs. NL: *t* = −0.89, *p* = 0.657).

#### Resting Metabolic Rate

**FIGURE 7:**
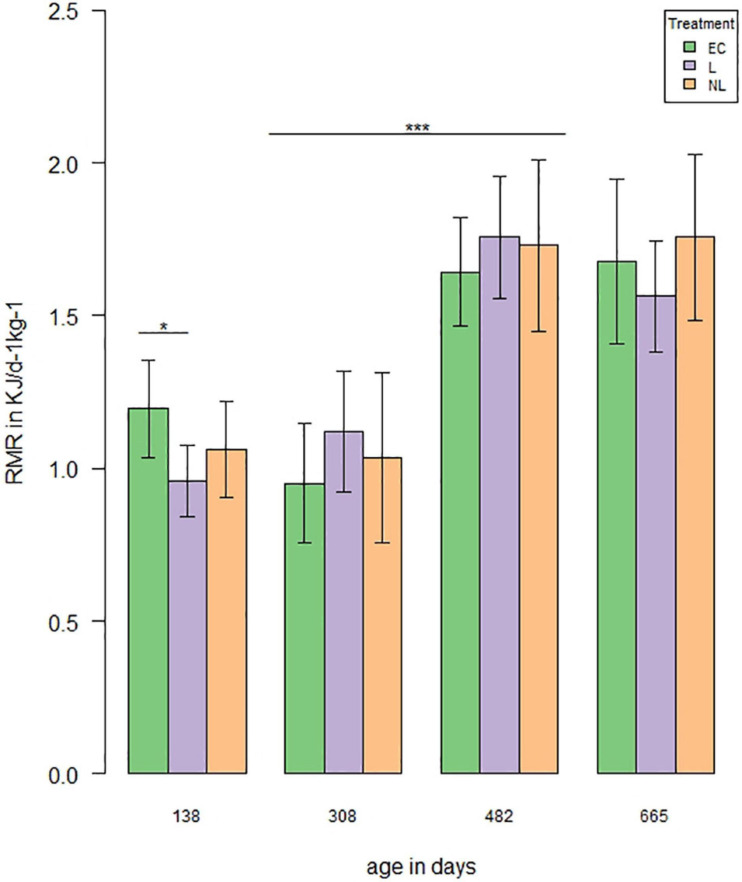
Resting metabolic rate (RMR) over time. RMR was measured four times (138, 308, and 482 days of age *n* = 48, 665 days of age *n* = 44). The analysis showed a treatment effect on RMR with an age of 138 days between EC and L mice (*t* = 2.58, *p* = 0.04). RMR increased significantly between the second and third measurement (|*t*| > 7.0, *p* < 0.001; *p < 0.05, ***p < 0.001).

A total of four measurements were performed to investigate the influence of learning treatment on RMR. When the mice were housed in the IC’s, the RMR differed in the GLMM *post hoc* comparisons between L and EC mice (Figure 7, first RMR measurement: *t* = 2.58, *p* = 0.04), while there was no difference between L and NL (*t* = −1.16, *p* = 0.25) or EC and NL (*t* = 1.43, *p* = 0.24). When the mice were housed outside the IC’s, no treatment effect was detected (GLMM, *post hoc* comparisons, second RMR measurement: L and EC: *t* = −1.08, *p* = 0.61; EC and NL: *t* = −0.24, *p* = 0.81; L and NL: *t* = 0.84, *p* = 0.61; third RMR measurement: EC and L: *t* = −0.92, *p* = 0.61; EC and NL: *t* = −0.84, *p* = 0.61; L and NL: *t* = 0.07, *p* = 0.94; fourth RMR measurement: EC and L: *t* = 0.003, *p* = 0.8, EC and NL: *t* = −0.68, *p* = 0.75, L and NL: *t* = −0.68, *p* = 0.75). While the RMR did not differ between the first two (GLMM, *t* = 0.42, *p* = 0.68) and last two measurements (GLMM, *t* = 0.53, *p* = 0.68), it increased significantly between the second and third measurement (GLMM, *t* = 7.0, *p* < 0.001).

### Effects on Sexually Selected Traits

#### Female Choice Tests

In the first female choice test, statistical analyses did not indicate a cage bias (GLMM, *t* = 1.03, *p* = 0.31), or an effect of male weight calculated as either body mass (GLMM, *t* = 0.40, *p* = 0.69), measured directly before the test, or mass rank within the trio of males (GLMM, *t* = −1.01, *p* = 0.32). Female mice preferred NL males over L males (GLMM, *post hoc* comparison, *t* = 2.47, *p* = 0.036, Table 2) and EC males (GLMM, *post hoc* comparison, *t* = 2.41, *p* = 0.036) but did not differentiate between EC and L males (GLMM, *post hoc* comparison, *t* = 0.14, *p* = 0.89).

**TABLE 2:** Effects of IntelliCage treatment on sexually selected traits.

Task	Behavioral response	EC	NL	L
Female choice I (age: 172 days)	% time spent with male	29.7 ± 5.0	**44.2 ± 5.2**	26.1 ± 5.1
Female choice II (age: 603 days)	% time spent with male	26.9 ± 8.7	35.7 ± 8.8	37.4 ± 8.7
Observations of agonistic behavior	# of fights involved	47.9 ± 5.8	42.8 ± 5.8	52.9 ± 5.8
	# of fights won	23.6 ± 4.2	22.7 ± 4.5	25.6 ± 2.9

*Estimates in bold font indicate a significant difference from other treatment levels.*

The second mate choice test likewise indicated no cage bias (GLMM, *t* = 0.49, *p* = 0.62) and no effect of body mass (GLMM, *t* = 0.55, *p* = 0.60) or mass rank within a trio (GLMM, *t* = 0.45, *p* = 0.66) on the choice of the females. In contrast to the first mate choice test, we found no indication for a preference of males of a specific treatment group (GLMM, *t* = 0.55, *p* = 0.58, Table 2).

#### Observations of Agonistic Behavior

Each cage of mice was observed for 26 h in which mice were involved in a mean number of 48 ± 4 fights. Mice of the L group were involved in fights significantly more often than the NL mice (GLM, *post hoc* comparison, *p* = 0.011) while no difference was found between NL and EC mice (Table 2). Additionally, heavier mice were more likely involved in fights (GLM, *z* = 3.94, *p* < 0.001). While heavier mice at the same time were more likely to win fights (GLM, *z* = 0.02, *p* = 0.034), no effect of treatment was found with regard to winning or losing fights (GLMM, *post hoc* comparisons, EC - L: *z* = −1.23, *p* = 0.434, EC-NL: *z* = −0.49, *p* = 0.875, L - NL: *z* = 0.71, *p* = 0.756).

### Effects on Longevity

#### Telomere Length

**FIGURE 8:**
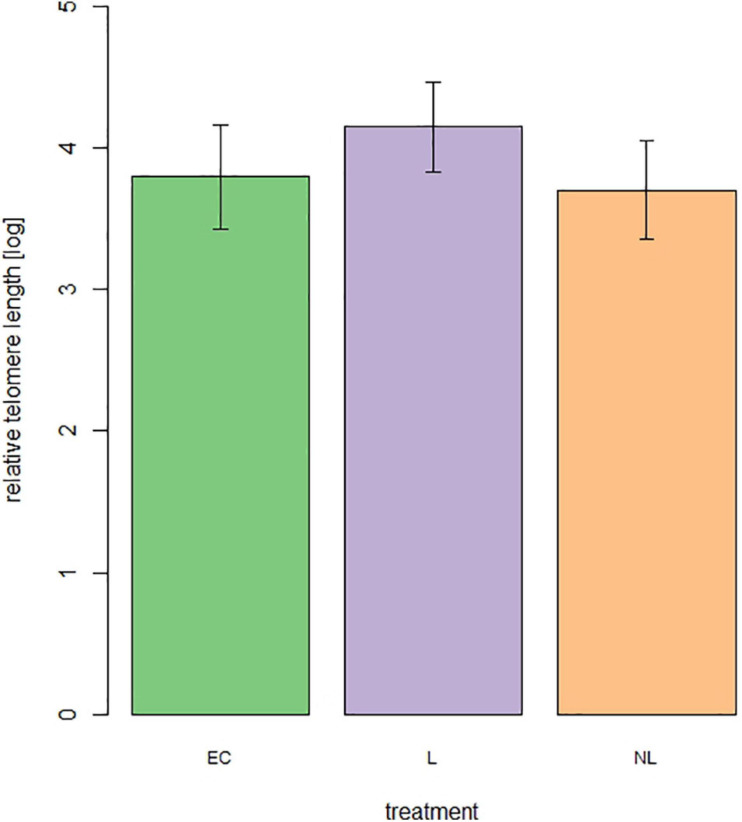
Relative telomere length of male mice at the age of 699 days (*n* = 44 mice, 15 = L, 14 = NL, 15 = EC).

For telomere length measurement 44 out of 48 mice were still alive. From these 44 mice 15 were L mice, 14 NL mice, and 15 EC mice. The treatment (Figure 8) did not influence the relative telomere length of the mice at an age of 699 days (linear model, adjusted *R*^2^ = 0.04, *F* = 1.84, *p* = 0.17).

#### Survival Analysis

**FIGURE 9:**
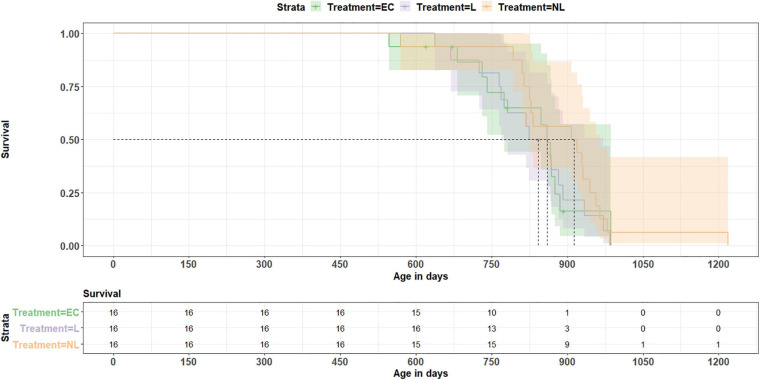
Survival analysis. The data show no treatment effect (EC = equipment control group, L = learner, NL = non-learner) on survival (*p* = 0.43). Dashed lines indicate median length of survival for each treatment group.

On average, the mice reached an age of 835 days. The shortest living mouse (EC) died at an age of 547 days, the oldest one reached an age of 1,218 (NL) days. Calculating a Coxph model, we investigated whether treatment or telomere length at the age of 699 days influenced longevity (Figure 9). All 44 mice which reached this age were included in the analysis. The model revealed no influence of the treatment on survival (EC vs. L: *z* = −0.6, *p* = 0.548; EC vs. NL: *z* = −1.8, *p* = 0.072; L vs. NL: *z* = −1.2, *p* = 0.231) and also no linkage between telomere length and survival (Coxph, *z* = 0.3, *p* = 0.780).

## Discussion

In this study we provide a unique observation of mice throughout their lives, from the juvenile stage to the natural death of the animals. By observing the mice during three consecutive phases, we obtained a lifetime data set. Our aim was to investigate if and how periods of cognitive stimulation affect animals on short term and over their lifetime. To produce cognitive stimulation, we used an automated IC system in which some mice in the social group were constrained by specific learning paradigms to where they could access water. Based on these environmental differences, we investigated a multitude of behavioral and physiological traits throughout the animals’ lives as well as potential effects on longevity. Despite some problems with the automated testing system, the exposure of young mice to cognitive stimulation resulted in several immediate behavioral and physiological effects such as elevated foraging behavior, slower growth and a lower RMR. However, these effects did not manifest in long-term consequences. Re-exposing mice to new learning routines later in life did not result in similar learning success as in young mice. Accordingly, we also did not observe behavioral or physiological differences resurfacing during these later stages of cognitive stimulation. The explanation for no differences later in life could be that the second and third IC phases were much shorter compared to the first phase. Originally, it was planned to cognitively stimulate the L mice repeatedly, so as to induce a cognitively demanding life. Since the first phase resulted in a more obvious stimulation compared to the second and third phases, we assume that the first phase (while the mice were young) had a greater impact than the two shorter IC phases at later ages.

Several differences between the groups emerged during the first IC phase, when L mice were cognitively stimulated and most successful in learning. The L mice showed a higher foraging behavior in this phase, possibly resulting from searching for the correct drinking corner, while the NL and EC mice had access to water in all corners at all times. In addition, the L mice had less time to drink per visit than the EC and NL mice. The doors within the correct corners opened after a nosepoke for L mice for 8–10 s (depending on the IC program). For EC and NL mice the doors in all corners opened for 15 s after a nosepoke. To drink sufficiently, the L mice therefore had to visit the correct corners more often. The higher foraging behavior might have contributed to the slower weight gain compared with NL mice. However, the body mass of EC mice was comparably lower as for the L mice. At this time of life, the individual weights of mice are most variable (Eisen, 1976) and experiencing early life stress (Clutton-Brock et al., 1992; Lindström et al., 2005; Douhard et al., 2013) as well as the development of increased cognitive capacity (Kotrschal et al., 2015) have been shown to be accompanied by slower growth rates. L and EC mice were repeatedly placed on test set-ups by tail handling, which is known to increase stress and anxiety (Gouveia and Hurst, 2013; Ghosal et al., 2015). Since the NL mice were the least affected by experimental procedures, had unrestricted access to water, and were not cognitively stimulated, they likely had the most energy available to invest in growth, which resulted in a higher body mass gain. No difference in weight gain between the L and EC mice might indicate that the experienced stress in these two groups of mice might have been more influential as compared to a direct influence of the cognitive stimulation on the growth rate of the mice. The failure to find the same difference in older mice can be due to the much shorter duration of the IC phases 2 and 3.

By measuring the RMR, we examined a further physiological parameter and assumed that cognitively stimulated mice might have a higher energy turnover compared to non-stimulated mice. The RMR was measured at four different time points but differences between treatments were only found for the first measurement, when the mice were housed within the ICs. The L mice had a lower RMR compared to the EC mice, while EC and NL mice did not differ from each other. RMR is often referred to as the energetic cost of an organism’s self-maintenance (McNab and Eisenberg, 1989; Speakman et al., 2004). Schubert et al. (2008) showed that increased foraging behavior resulted in reduced body mass and RMR. The L mice in our study also showed higher levels of foraging behavior and reduced body mass and RMR. The reduced RMR could be explained by an energy-saving strategy (Moe et al., 2007; Mathot et al., 2009), since a low RMR is commonly associated with demanding life-stages (e.g., under environmental stress or during reproduction). However, the energy-saving strategy did not seem to be necessary for a longer period of time, as the RMR differences were no longer present in the following measurements. This could be due to the fact that there were no more differences in foraging behavior or body mass. The underlying reason may be that the L mice were no longer successfully learning in the following IC phases. Interestingly, the RMR in all treatments increased with age.

Aging is characterized by declines in all physiological processes and concomitant changes in body composition. Age-related changes in body composition and physiological function are commonly reflected in a reduced metabolic rate in older individuals (Tzankoff and Norris, 1977; Piers et al., 1998). However, mixed patterns have been described depending on the species observed, the type of metabolic rate and the environment investigated (Elliott et al., 2015). Effects of the physical environment, the sex and the food availability have not been investigated systematically yet and the mechanism of metabolic aging is not well understood yet (Moe et al., 2007; Elliott et al., 2015). Here, we found an increase in metabolic rate with age, comparable to what has been found in rats from an age of 18 months on (McCarteer and Palmer, 1992). Nevertheless, the increase in RMR from 308 to 482 days of age is quite steep and we cannot exclude the possibility that other, external influences had an effect on the measurements. For example, while room and experimental conditions during the RMR measurements were kept constant, the animals had been relocated to another holding facility in between. We made sure that temperature, humidity and air pressure were comparable but it might have been that mice reacted to factors that escaped our perception.

In addition to the influence of cognitive stimulation on physiological processes, we investigated whether and how cognitive stimulation affects male attractiveness and dominance. Some studies showed that females of different species chose based on morphological features (Bischoff et al., 1985; Mateos and Carranza, 1995; Kodric-Brown and Nicoletto, 2001), scent marks (Ramm et al., 2008) or by cognitive performance [reviewed in Boogert et al. (2011)]. The question in our study was, whether or not male mice which were cognitively stimulated were also preferred by females. On the contrary, our results showed that female mice preferred NL mice, which were never cognitively stimulated like L mice or additionally stressed by tail handling like EC and L mice. Female mice did not differentiate between L and EC mice. Since females’ choices were not influenced by the body mass of the males and they could not directly assess the males’ cognitive abilities in the mate choice test, other factors have to be considered. Several volatile substances in male mice urine have been shown to provide mate assessment signals (Stopka et al., 2012). Scent production is known to be associated with male social status, stress level, and other factors of the physical environment, thus allowing females to assess male quality (Novotny et al., 1985, 1990). The attractiveness of the scent mark is influenced by the quality and quantity of its compounds (Drickamer, 1992; Zala et al., 2004). Gosling et al. (2000) showed that high intensities of scent marks are costly in terms of body mass loss and loss of dominance status. As our L mice already had higher energy demands (higher activity, reduced body mass, and RMR), there may have been less energy available for the production of costly signals. The EC mice may have been stressed by the additional handling by being placed on test set-ups through tail handling without having the opportunity to escape this situation (like the L mice) which may also have had a negative effect on the chemical signals produced. Thus, the L mice and EC mice may have had a lower quality and/or quantity of scent marks and were therefore less attractive for female mice.

In our study the female mice only differentiated between the treatment groups during the first FC test. There was no female choice during the second FC. One explanation could be that there might not have been a detectable difference between treatments from the perspective of the females. This would be plausible as the L mice did not learn the tasks in the second and third IC phase which was accompanied by the fact that the treatments did not result in measurable physiological differences. Therefore, the females may not have been able to distinguish between the groups. Or the male mice were generally unattractive because of their age (592 days) regardless of the treatment. The post-reproductive phase appears to be strain-dependent and begins in wild mice by reduced fertilization from 570 days [reviewed in Brust et al. (2015)]. If this is also true for C57BL/6J mice, old age could possibly influence the female’s choice during the second FC test. Finally, future experiments where females can directly assess the learning capabilities of male mice are needed to more clearly assess the validity of the influence of cognitive stimulation on female choice.

One further important factor in increasing attractiveness is the social rank of a male. But unfortunately, the social rank of the male mice in our study was not investigated during the first IC phase and the first FC test where the females differentiated between the groups. The observations recorded later showed that none of the three treatment groups differed with regard to winning or losing fights. However, the L mice were overall involved in more fights. Whether they also initiated them and thus have a greater potential for aggression cannot be determined with our data. However, it should be noted that fighting behavior was rare and only observed during cage cleaning. There was no need to remove individual mice from the groups at any time due to aggressive behavior and resulting injuries. We would argue that the relatively small environment and large number of males may simply not enable building territories and rank hierarchies.

In addition to physiological and fitness related costs of cognition we examined the influence of repeated cognitive stimulation on longevity. Therefore, we investigated telomere lengths in aged mice (699 days). It is known from the literature that the length of telomeres is associated with disease, loss of cognitive abilities, and longevity (Blasco et al., 1997; Lee et al., 1998; Rudolph et al., 1999; Cawthon et al., 2003; Benetos et al., 2004; Martin-Ruiz et al., 2006; Yaffe et al., 2011). Repeated cognitive stimulation might have led to higher energetic costs and compensation of these costs in terms of a reduced investment in other energetically costly physiological processes, which might reflect in shorter telomere lengths and lifespans. However, we did not find such an effect in our mice. Even though mice of the EC and L group both showed lower growth rates during the first IC phase, they caught up later in life, as it has been observed numerous times across many species under improving conditions (Metcalfe and Monaghan, 2001). Similarly, investment in other physiological processes may have been postponed rather than fully neglected if they became apparent due to the different environments experienced, especially during early life in the mice of this study. Still, both, early conditions experienced in life as well as compensation strategies (Metcalfe and Monaghan, 2001; Burton and Metcalfe, 2014) can hold long-term costs. The fact that we could not identify such costs may lie in the continuous availability of *ad libitum* food throughout the mice’s life, which may have helped our animals to successfully catch up without suffering from long term consequences of the experienced early environment. In addition, one could argue that introducing cognitive tasks into the life of laboratory mice serves as cognitive enrichment, i.e., the possibility to use evolved cognitive skills to solve problems and control aspects of the environment (Clark, 2017). Cognitive enrichment is known to increase neuroplasticity properties and increase neural connectivity [reviewed in Petrosini et al. (2009)] and it possibly reduces boredom related abnormal behavior. This seemingly protects against the development of age-associated cognitive decline and functional impairments even in the presence of brain pathologies in laboratory rodents (Stern, 2002; Milgram et al., 2006).

As already mentioned earlier, during the first IC phase, the cognitive stimulation induced observable changes in behavior and physiology, while during the second and third IC phase, the L mice did not learn successfully within the IC. In addition, the L mice had to solve fewer cognitive tasks in the second and third IC phases compared to the first IC phase. Therefore, we might assume a cognitively demanding start in life rather than a cognitive demanding life in our study. This was reflected in our results. While differences in behavior and physiological processes were detected in young mice during the first IC phase, these differences were no longer present in the following measurements. Therefore, it is not surprising that no effects on longevity could be detected. Both the telomere length and the survival analysis showed no treatment differences.

Previous studies have already demonstrated that the IC is a useful tool for investigating learning behavior (Galsworthy et al., 2005; Mechan et al., 2009; Krackow et al., 2010; Endo et al., 2011; Voikar et al., 2018). As mentioned earlier, the L mice reached the learning criterion in the IC tasks only during the first phase. In the following phases, however, the cognitive stimulation was not successful. The tasks during the first IC phase were supposedly easy to learn and similar to those of other IC studies. In contrast, the tasks during the second and third phase were chosen to be more complex and were conducted at an older age of the mice. We exclude that the L mice had severe age-related cognitive decline, as we were able to show that the L mice in the BM were also learning at the age of 573 days. This indicates that aged mice indeed are capable of showing reasonable performances which is also in accordance with literature data (Mechan et al., 2009). In addition, we retrospectively noticed that although the mice received an airpuff as punishment for visiting the wrong corner they still drank in these corners. This was unexpected as we assumed that they would avoid the airpuffs as is known from other experiments. So most likely they did not perceive the airpuff as a severe punishment, habituated after repeatedly being exposed to this stimulus, and accordingly developed a more relaxed attitude. Mice are known for notoriously using alternative strategies in tasks laid out by humans (Habedank et al., 2021). For example, in cognitive testing the use of semi-successful but often simpler strategies is common but often corrected for by the experimenter right away. Using an automated testing system prevented us from quickly noticing that the mice adopted an alternative strategy to solve some of the tasks and accordingly we could not adjust the experiment. Working with an automated system is advantageous in terms of not stressing the animals, for example by separating them from their social group or being in contact with a human experimenter on a regular basis. At the same time it leads to delayed feedback making it harder to detect flaws in the setup and execution of experiments, especially while these are running.

We demonstrated that the IC system, as an automated and home-cage based test system, is a useful method to keep mice with different treatments in one social group. Furthermore, the system worked extraordinarily well even for group housed male mice, which are often housed singly to prevent overt aggression. Hence, our setting supports calls of the current legislation (e.g., EU directive 2010/63) to house mice in social groups whenever possible. To keeping the mice within the ICs, they were well habituated to the test procedure and it was possible to observe the mice in their natural active phase. While the mice had to be handled for all tests outside the IC system, which is presumably associated with stress, the IC experiments themselves could be carried out without disturbance by the experimenter. We also showed that using the same group of mice repeatedly allowed us to perform experiments without treating laboratory mice as disposable goods like it is still common practice (Brust et al., 2015). This approach also reduces the overall number of experimental animals and is in accordance with the 3Rs (reduce, refine, and replace). In summary our data suggest that the IC system is a highly useful tool to conduct unique home-cage based long-term studies in social settings. In addition, our study showed that cognitive stimulation induces reversible short-term changes in behavior and physiology. We could not detect any long-term effects on behavior or physiology. Similarly, we also did not find persisting effects on sexually selected traits such as dominance or mate choice and no effects on longevity. To our best knowledge, our study provides the first unique long-term data set from male mice and we hope that this will guide future sustainable and responsible studies in laboratory animal science.

## Data Availability Statement

The raw data supporting the conclusions of this article will be made available by the authors, without undue reservation.

## Ethics Statement

The animal study was reviewed and approved by LAVES, license 33.9-42502-05-14A430.

## Author Contributions

PK, AG, LL, and VB: conceptualization. PK, JT, and EH: conducting the experiments. PK, AG, MB, and VB: data analyzing and visualization. LL: project administration and supervision. PK, AG, MB, VB, and LL: article writing, review, and editing. All authors contributed to the article and approved the submitted version.

## Conflict of Interest

The authors declare that the research was conducted in the absence of any commercial or financial relationships that could be construed as a potential conflict of interest.

## Publisher’s Note

All claims expressed in this article are solely those of the authors and do not necessarily represent those of their affiliated organizations, or those of the publisher, the editors and the reviewers. Any product that may be evaluated in this article, or claim that may be made by its manufacturer, is not guaranteed or endorsed by the publisher.
