# Supplementary material for: Lifetime Observation of Cognition and Physiological Parameters in Male Mice
Source: Front Behav Neurosci. 2021 Sep 1;15:709775. doi: 10.3389/fnbeh.2021.709775 (PMC8442583; doi:10.3389/fnbeh.2021.709775)
Supplement: Supplementary file 1 [file Data_Sheet_1.pdf]

Supplementary Material

1 Supplementary Information

The IC is an automated and home-cage based test system in which various cognition tasks were applied to individual animals with minimal invasion by experimenters. Water was available in eight dispensers arranged in the four cage corners. Each corner contains a presence sensor, one RFID antenna, an airpuff valve for a mild punishment (0.5 bar), two water dispensers, two doors and two nosepoke sensors. The access to single water dispensers can be granted to individual mice identified via the RFID antenna. In the following IC task descriptions the following definitions apply:

Correct corner = Water was available, the doors can be opened by performing a nosepoke on the nosepoke sensors.

Incorrect corner = The doors were not opened by performing a nosepoke on the nosepoke sensor. In some IC tasks there was also an airpuff following a nosepoke performed in the wrong corner.

Correct side/door = Water was available on one water dispenser (either the right or the left water bottle) within the corner, the door can be opened by performing a nosepoke on the nosepoke sensor on this side.

Incorrect side = The door to the water bottle was not opened by performing a nosepoke on the nosepoke sensor. In some IC tasks there was also an airpuff following nosepoke performed on the wrong side.

Visit = One mouse entered the corner and was detected by both, the presence sensor and the RFID antenna. Visits where at least one nosepoke was performed are considered as a trial, except when described otherwise.

1.1 IntelliCage Tasks

Cornerlearning 1

Each mouse was assigned one correct corner with both sides correct. Every day, the correct corner became incorrect and another corner became the correct corner. The order of the corner changes was corner 1 -> corner 3 -> corner 2 -> corner 4 -> corner 1 etc. Entering a corner registered a visit and the

L-mice had the opportunity to perform a nosepoke in the correct corner at either of the two nosepoke sensors to open the doors for 10 seconds. The doors could be opened repeatedly during a visit in a correct corner, but 20 seconds after the visit registration, a mild airpuff was released. A visit in the incorrect corners had no further effect. Duration of the task: 13 days and 21:52:59 hours.

Cornerlearning 2

Each mouse was assigned one correct corner. On day three, four, five, seven and ten the former correct corner became incorrect and another corner was assigned as correct. The order of the corner changes was corner 1 -> corner 3 -> corner 2 -> corner 4 -> corner 1 etc. Entering a corner registered a visit and the L-mice had the opportunity to perform a nosepoke in the correct corner at either of two nosepoke sensors to open the doors for 8 seconds. The correct doors could be opened repeatedly during a visit, but 15 seconds after the visit registration, a mild airpuff was released. A visit in the incorrect corners also resulted in a mild airpuff punishment. Each visit (irrespective of performed nosepoke) is considered as a trial. Duration of the task: 12 days and 20:39:26 hours.

Shuttling 1

Each mouse was assigned one correct corner. After each drinking event in the correct corner the current correct corner became incorrect and the opposite corner became correct. For example, corner 1 became incorrect after a drinking event and corner 3 became correct and vice versa, while corner 2 and 4 were permanently incorrect. The mice had to shuttle between corner 1 and 3. After four, seven and nine days since the start of Shuttling 1, a diagonal corner side change occurred. For example, the correct corners 1 and 3 became incorrect and corner 2 and 4 became correct. Thus, the mice had to shuttle between corner 2 and 4. Entering a corner registered a visit and the L-mice had the opportunity to perform a nosepoke in the correct corner at either of the two nosepoke sensors to open the doors for 10 seconds. The correct doors could only be opened once per visit and 15 seconds after the visit registration, a mild airpuff was released. A visit in the incorrect corners had no further effect. Duration of the task: 11 days and 19:31:40 hours.

Shuttling 2

Each mouse was assigned one correct corner. After each drinking event in the correct corner the current correct corner became incorrect and the opposite corner became correct. For example, corner 1 became

incorrect after a drinking event and corner 3 became correct and vice versa, while corner 2 and 4 were permanently incorrect. The mice had to shuttle between corner 1 and 3. After day one, three, and five since the start of Shuttling 2, a diagonal side change occurred. For example, the correct corners 1 and 3 became incorrect and corner 2 and 4 became correct and now the mice had to shuttle between corner 2 and 4 after each drinking event. Entering a corner registered a visit and the L-mice had the opportunity to perform a nosepoke in the correct corner at either of the two nosepoke sensors to open the doors for 10 seconds. The correct doors could only be opened once per visit and 15 seconds after visit registration, a mild airpuff was released. Each incorrect visit resulted also in a mild punishment. Each visit (irrespective of performed nosepoke) is considered as a trial. Duration of the task: 6 days and 21:10:06 hours.

Complexshuttling

All L mice were assigned one correct corner but they had access to water in all four corners. All doors could only be opened once per visit for 8 seconds. In three out of four corners (incorrect corners), the L mice received a mild airpuff by entering the incorrect corners. Within the correct corner, drinking was possible without receiving an airpuff. In this correct corner, one side was set as incorrect and the other side was set as correct. By each drinking event on the incorrect side within the correct corner, the current correct corner became incorrect and the opposite corner became correct. In addition, a diagonal change occurred daily. If the mice first had to shuttle between corner 1 and corner 3, they then had to shuttle between corner 2 and 3 after the diagonal side change and vice versa. Duration of the task: 13 days and 20:29:50 hours.

Clockwise 1

At the beginning of Clockwise 1, for all mice corner 1 was set as the correct corner. After each drinking event the current correct corner became incorrect and the next corner clockwise became correct. Entering a corner registered a visit and the L-mice had the opportunity to perform a nosepoke in the correct corner at the nosepoke sensors to open the doors for 10 seconds. The correct doors could be opened repeatedly during a visit, but 15 seconds after the visit registration, a mild airpuff was released. All other corners were incorrect and a visit with nosepoke on the nosepoke sensor resulted in a mild punishment. Duration of the task: 10 days and 21:31:48 hours.

Clockwise 2, 3, 4, 5

Each mouse was assigned one correct corner. After each drinking event the current correct corner became incorrect and the next corner clockwise became correct. Entering a corner registered a visit and the L-mice had the opportunity to perform a nosepoke in the correct corner at the nosepoke sensors to open the doors for 10 seconds. The correct doors could be opened repeatedly during a visit, but 15 seconds after the visit registration, a mild airpuff was released. All other corners were incorrect and a visit resulted in a mild punishment. Duration of the Clockwise 2: 9 days and 00:43:19 hours; Clockwise 3: 8 days and 20:31:53 hours; Clockwise 4: 5 days and 21:23:17 hours; Clockwise 5: 10 days and 01:40:10 hours.

Clockwise 6

All mice had access to water in all four corners. In three out of four corners, the mice received a mild airpuff by each visit (set as wrong corners). Within the correct corner, drinking was possible on both dispensers. Each mouse was assigned one correct corner. After each drinking event within the correct corner, this corner became wrong and the next corner clockwise became correct. Entering the correct corner registered a visit and the L-mice had the opportunity to perform a nosepoke in the correct corner at the nosepoke sensors to open the doors for 10 seconds, but 15 seconds after the visit registration, a mild airpuff was released. Duration of the task: 2 days and 22:34:30 hours.

Complexclockwise 1 and 2

All L mice were assigned one correct corner but they had access to water in all four corners. All doors could only be opened once per visit for 8 seconds. In three out of four corners (incorrect corners), the L mice received a mild airpuff by entering an incorrect corner. Within the correct corner, drinking was possible without receiving an airpuff. But a drinking event in the correct corner on the incorrect side leads to a corner change. The current correct corner became incorrect and the next corner clockwise became the correct corner. Entering the correct corner registered a visit and the L-mice had the opportunity to perform a nosepoke in the correct corner at the nosepoke sensors to open the doors for 8 seconds, but 15 seconds after the visit registration, a mild airpuff was released. Example: Corner 1 is the correct corner and the water dispenser on the right was set as the incorrect side, the left side was set as the correct side. A drinking event on the correct side within the correct corner had no further effect i.e., the mice had to reenter the same corner for the next drinking event. A drinking event on the incorrect side within the correct corner leads to a corner change. The current correct corner became

incorrect and the next corner clockwise became correct. Duration of Complexclockwise 1: 9 days and 23:13:39 hours; Complexclockwise 2: 1 days and 21:46:37 hours.

Anticlockwise 1

All mice were assigned one correct corner. After each drinking event the current correct corner became incorrect and the next corner anticlockwise became correct. Entering a corner registered a visit and the L-mice had the opportunity to perform a nosepoke in the correct corner at the nosepoke sensors to open the doors for 10 seconds. The correct doors could be opened repeatedly during a visit, but 15 seconds after the visit registration, a mild airpuff was released. All other corners were incorrect and a visit with a nosepoke on the nosepoke sensor resulted in a mild punishment. Duration of the task: 13 days and 21:28:39 hours.

Anticlockwise 2 and 3

All mice were assigned one correct corner. After each drinking event the current correct corner became incorrect and the next corner anticlockwise became correct. Entering a corner registered a visit and the L-mice had the opportunity to perform a nosepoke in the correct corner at the nosepoke sensors to open the doors for 10 seconds. The correct doors could be opened repeatedly during a visit, but 15 seconds after the visit registration, a mild airpuff was released. All other corners were incorrect and a visit resulted in a mild punishment. Duration of Anticlockwise 2: 19 days and 03:08:53 hours; Anticlockwise 3: 12 days and 19:56:29 hours.

Complexanticlockwise 1 and 2

All L mice were assigned one correct corner but they had access to water in all four corners. All doors could only be opened once per visit for 8 seconds. In three out of four corners (incorrect corners), the L mice received a mild airpuff by entering the incorrect corners. Within the correct corner, drinking was possible without receiving an airpuff. But a drinking event in the correct corner on the incorrect side leads to a corner change. The current correct corner became incorrect and the next corner anticlockwise became the correct corner. Entering the correct corner registered a visit and the L-mice had the opportunity to perform a nosepoke in the correct corner at the nosepoke sensors to open the doors for 8 seconds, but 15 seconds after the visit registration, a mild airpuff was released. Example: Corner 1 is the correct corner and the right water dispenser was set as the incorrect side, the left water dispenser was set as the correct side. A drinking event on the correct side within the correct corner had

no further effect. The mice had to reenter this correct corner for a next drinking event. A drinking event on the incorrect side within the correct corner led to a corner change. The current correct corner became incorrect and the next corner anticlockwise became correct. Duration of Complexanticlockwise 1: 11 days and 21:53:07 hours; Complexclockwise 2: 8 days and 00:20:07 hours.

1.2 IntelliCage foraging behavior

The daily number of licks per mouse are measured by the IntelliCage system and are shown in figure S1 for the IntelliCage experiment phases. Contrary to the number of corner visits mice make each day, the licks are directly coupled to water intake, therefore showing that the mice roughly drank a similar amount of water each day. Note that since mice vary in the amount of water intake per lick, we cannot accurately compare the water intake between conspecifics.

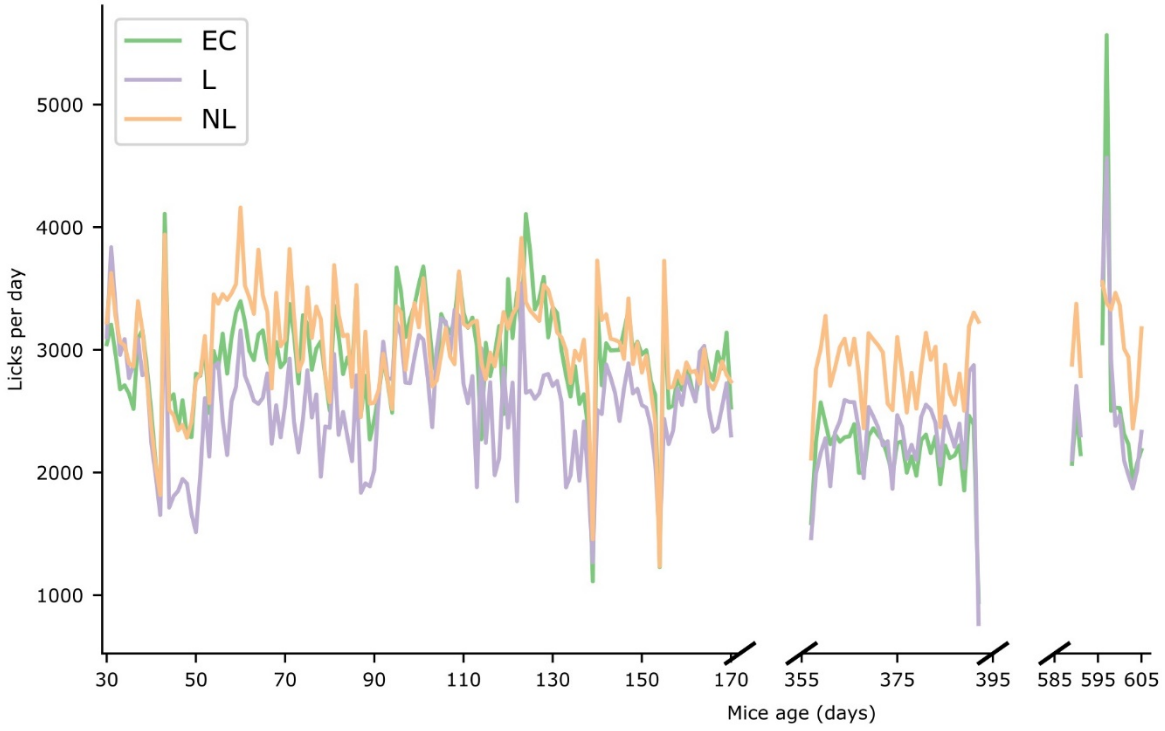

Figure S1: Daily water bottle licks during the three IntelliCage phases. The x-axis represents the age of the mice (in days). The average value of the three different treatment groups are shown for the three IntelliCage phases.

2 T-Maze

T-Maze is based on associative learning. A neutral stimulus acquires a new meaning because this stimulus becomes associated with a reward. The T-maze set-up (figure S2) in our study consisted of a long corridor (11 cm wide, 32 cm long) from which two corridors branched off in a T-shape (11 cm long, 75 cm long). One of the two corridors was the target corridor and connected by a tube to the IC. Access to the IC was used as a reward. The other corridor was the incorrect one without a connection to the IC. One corridor was marked inside on the walls with a black and white vertically striped pattern. The other corridor was marked with a black and white longitudinal striped pattern (stripe thickness: 1 cm). The position of the correct and incorrect corridor was pseudorandomized. It was ensured that the same number of changes of the correct corridor occured in each animal. This should ensure that the L mice selected the correct corridor based on the pattern and not on the position (left or right). Light barriers were located in all corridors to detect the movement of the mice. In addition, there were doors at the ends of the corridors that were controlled by light barriers. If a mouse broke through the light barrier of the door in the correct corridor, it opened and the mouse could leave the T-maze and enter the IC. There was no possibility to leave the T-maze through the incorrect corridor. A start box, where the mice were placed at the beginning of the test, was connected to the long corridor. After one minute, the door of the start box opened and the mouse could enter the T-maze. On the first day, the mice were habituated to the T-maze by giving them five minutes after leaving the start box to explore the T-maze and enter the IC out of the T-maze. When the mice did not return to the IC on their own within this time, they were led into the correct corridor by closing the incorrect corridor. This was to ensure that all mice knew the possibility of returning to the IC before the start of the experiment. Two habituation runs were performed per animal. The test trials were carried out on the following four days. On each test day, six trials were performed per animal. Each trial began with the one-minute start phase in the start box. After opening the door to the long corridor, the mice could enter the T-maze and choose one of the two corridors at the end of the T-maze. If the mouse chose the correct corridor, it remained in the IC until its next trial started. If it chose the incorrect corridor, it was placed in a separate cage until its next trial started. During all trials, the running behavior of the mice was noted (correct / incorrect). Furthermore, the time was measured that the animals needed to return to one of the corridors or to the IC after leaving the start box. After each trial of a L mouse, the door to the IC was closed. The matched EC mouse were then placed first for one minute in the start box and then into the T-maze for the time it took the L mouse to leave the T-maze without having the possibility to leave the T-maze itself. After each trial, the apparatus was cleaned with 70% ethanol. At the start of the T-maze test, the mice were

111 days old. The illumination of the test set-up was 10-20 lux. The room temperature was 23.4 +/- 0.9 °C and the humidity was 38.8 % +/- 4.5 %. Data acquisition was performed by the experimenter through direct observation in addition to the time measured by the light barrier system.

It was not feasible to condition the mice. In some cases, they remained in the T-maze for several minutes without showing any signs of wanting to leave and had to be guided by the experimenter to the correct passage and into the tube to the IC. Even after the T-maze was supposedly made more aversive (stronger lighting, wet corridors), the mice remained in the T-maze. Thus, it was decided to terminate the experiment without being able to determine the cognitive performance of the L mice.

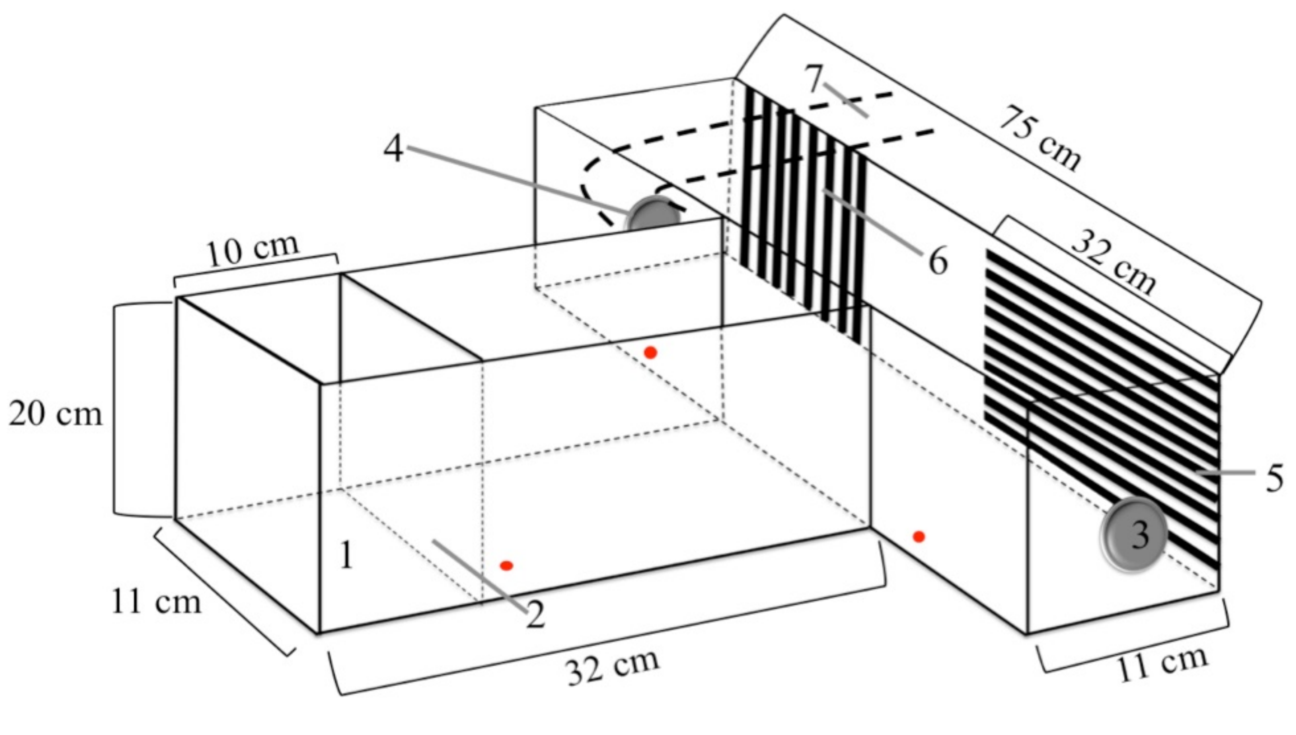

Figure S2: T-maze set-up. 1: Start box, 2: Door of the start box, 3: Inactive door of the incorrect corridor, 4: Active door of the correct corridor, 5: Marking of the incorrect corridor, 6: marking of the correct corridor, 7: Tube to the IC, Red dots: Positions of the light barriers.

3 Tone Conditioning

The tone conditioning test was based on associative learning. In this experiment, the mice were supposed to associate an acoustic signal with a food reward. The test set-up was a standard empty cage

(L: 36 cm; W: 26 cm; H: 15 cm) with a Plexiglas tube (diameter: 4 cm; L: 7.5 cm) attached to its side. The tube was equipped with a light barrier and a loudspeaker for acoustic signals. At the end of the tube was a feed mill filled with golden millet. The set-up is operated by a technical unit. A clock was integrated into this, which should enable the automated start of the test. The experiment comprised eight sessions. Several trials take place within a session. A trial consisted of an acoustic signal paired with the start of the feed mill. After the acoustic signal, the time until the light barrier was interrupted was measured. Only after interruption of the light barrier, another acoustic signal with feed delivery could take place. The timing of the acoustic signals was random, but always dependent on the condition that the light barrier was interrupted between two acoustic signals. There were longer breaks between the different sessions. During the experiment, water was freely available to the mice. Food was not additionally provided. Mice were placed in the set-up 4.5 h before the actual start of the test to prevent food intake immediately before the experiment. The experiment started 0.5 h before the dark cycle and ended 1 h before the light cycle (12.5 h). During this time, several sessions occurred in random order. The total number of in-session trials varied from mouse to mouse because a new trial occurred only when the mice between acoustic signals interrupted the light barrier. After each run of the L mice, a run was performed with the matched EC mice. For the EC mice, the determined amount of millet eaten by the matched L mouse was added directly to the tube. The set-up was cleaned before and after each test with 70% ethanol. The experiments were performed at a temperature of 24.21 +/- 0.4 °C and a humidity of 53.7 +/- 6.2 %. At the beginning of the test, the mice were 118 days old. Due to technical difficulties, the data was not reliably recorded. As a result, a meaningful statistical evaluation is not possible.

4 Labyrinth Experiment

For an additional spatial learning test a labyrinth test was carried out. At this time the mice were 633 days old. The labyrinth consisted of a white square base (Plexiglas; 110 cm x 39 cm) with pluggable walls (Plexiglas; 14.5 high) to build various corridors. The target was connected by a Plexiglas tube to a cage containing bedding from the home cage. At the beginning of the test, a mouse of the L group was placed in the start area (opposite of the target). For evaluation, the time required to reach the target was measured. In addition, the errors made by the L mice were counted. An error was counted as soon as a mouse entered with all paws a wrong corridor or turned around out of the right corridor. If a mouse did not reach the target within five minutes, it was guided through the labyrinth to the target. After

reaching the target cage the mice were placed in their home cage. The matched mouse of the EC group was placed in the labyrinth (at any place of the labyrinth) for the same time as the L mouse needed without the possibility to leave the labyrinth on its own. Before and after each trial the labyrinth was cleaned with ethanol (70 %). Two trials per mouse were performed on each day, with a 60 minutes inter-trial interval. In total, the tests were performed on four consecutive days. The room temperature was 20 °C +/- 1.5 °C and the humidity was 40 % +/- 7.5 %. Data acquisition was performed by the experimenter through direct observation.

The number of errors was analyzed in a Poisson GLMM (R-package lme4) including errors and trial as fixed effects and animal ID as random effect. The time to escape from the labyrinth was analyzed with a Friedman test (time to escape and trial = fixed effects and animal ID as random effect). The results showed an increase in errors made (GLMM, $p < 0.001$, figure S3). Whereas the time needed for the mice to leave the maze decreased (Friedman test, $p < 0.001$, figure S4). The results indicate that the motivation to leave the maze was present, but the way to the target was either not memorized or priority was given to exploratory behavior. Therefore, it was not unequivocally clear whether or not the L mice successfully learned the task.

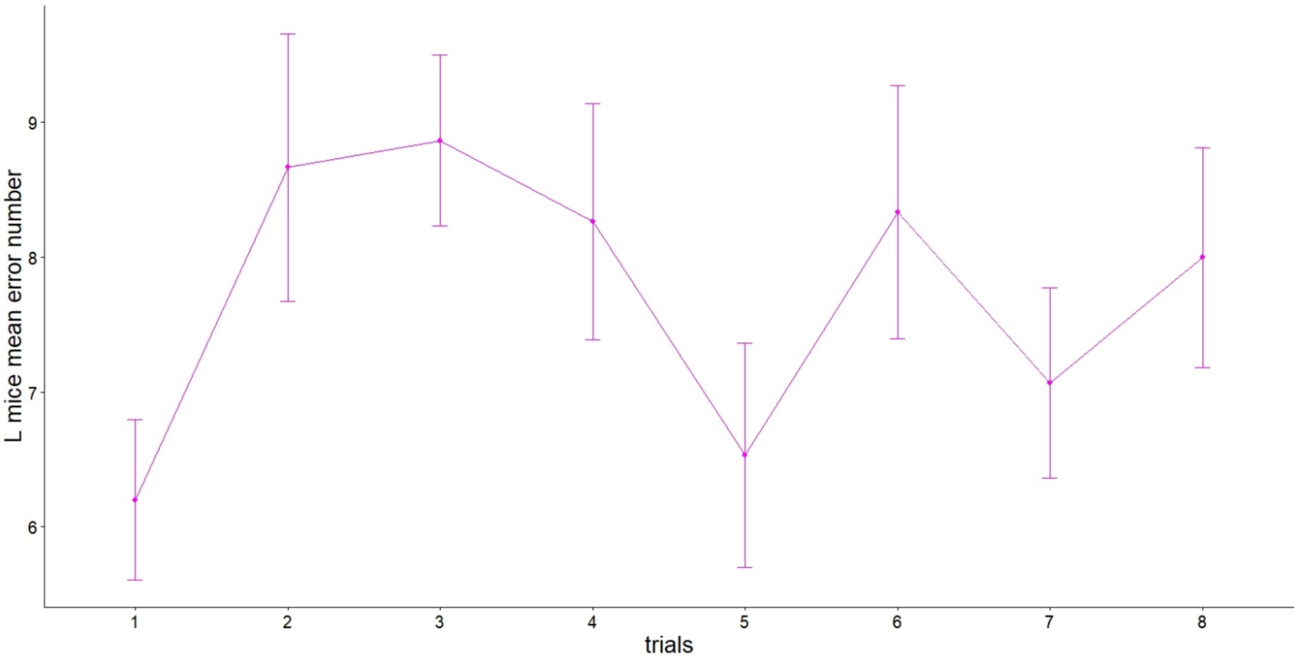

Figure S3: Number of errors made in the labyrinth (n = 14).

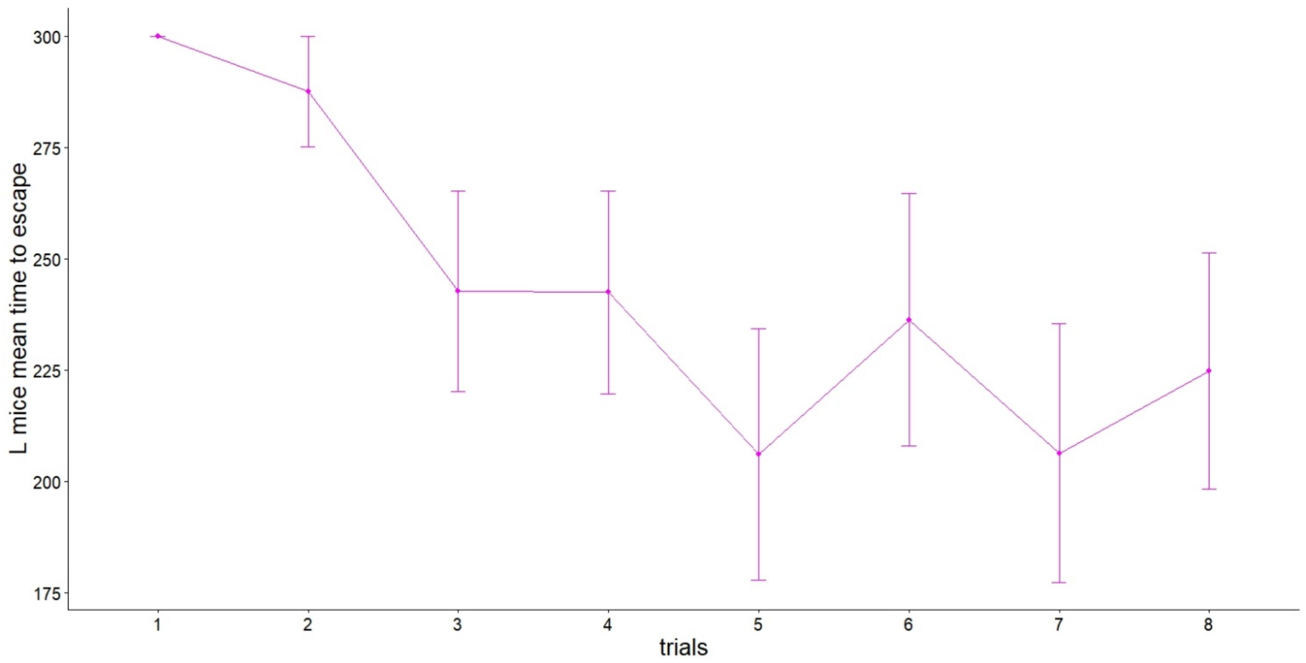

Figure S4: Time to escape from the labyrinth (n = 14).

5 Tabular time course of the experiments

Table S1: Timetable of experiments.

Experiment	Time of experiment in days	Age in days *
IntelliCage Phase 1	142	30
Barnes Maze Test	5	92
T-Maze **	5	111
Tone Conditioning **	32	118
Resting Metabolic Rate 1	8	138
Female Choice Test 1	37	152
Resting Metabolic Rate 2	8	308

Move	1	325
IntelliCage Phase 2	37	357
Resting Metabolic Rate 3	8	482
Barnes Maze Test 2	3	573
IntelliCage Phase 3	18	589
Female Choice Test 2	20	590
Labyrinth	5	633
Resting Metabolic Rate 4	8	665
Telomere Length Measurement	1	699

* at the beginning of the experiment

** not included in main paper
